# Measuring the exposure of Black, Asian and other ethnic groups to COVID-infected neighbourhoods in English towns and cities

**DOI:** 10.1007/s12061-021-09400-8

**Published:** 2021-09-03

**Authors:** Richard Harris, Chris Brunsdon

**Affiliations:** 1 School of Geographical Sciences, University of Bristol, University Road, Bristol, BS8 1SS UK; 2 National Centre for Geocomputation, Maynooth University, Maynooth, Co. Kildare Ireland

**Keywords:** COVID-19, Infections, Index of exposure, BAME groups, Ethnicity, Inequality, Health

## Abstract

Drawing on the work of The Doreen Lawrence Review—a report on the disproportionate impact of COVID-19 on Black, Asian and minority ethnic communities in the UK—this paper develops an index of exposure, measuring which ethnic groups have been most exposed to COVID-19 infected residential neighbourhoods during the first and second waves of the pandemic in England. The index is based on a Bayesian Poisson model with a random intercept in the linear predictor, allowing for extra-Poisson variation at neighbourhood and town/city scales. This permits within-city differences to be decoupled from broader regional trends in the disease. The research finds that members of ethnic minority groups can be living in areas with higher infection rates but also that the risk of exposure is distributed unevenly across these groups. Initially, in the first wave, the disease disproportionately affected Black residents but, as the pandemic has progressed, especially the Pakistani but also the Bangladeshi and Indian groups have had the highest exposure. This higher exposure of the Pakistani group is not straightforwardly a function of neighbourhood deprivation because it is present across a range of average house prices. We find evidence to support the view, expressed in The Doreen Lawrence Review, that it is linked to occupational and environmental exposure, particularly residential density but, having allowed for these factors, differences between the towns and cities remain.

## Introduction

COVID-19 is having a disproportionate and devastating impact on ethnic minority communities. Not only are Black, Asian and minority ethnic people dying at a disproportionate rate, they are also overexposed to the virus and more likely to suffer the economic consequences (*The Doreen Lawrence Review*, 2020: opening sentences of the Executive Summary).

This paper looks at the exposure of Black, Asian and minority ethnic (BAME) groups to neighbourhoods of greater than expected rates of COVID-19 infection, in English major towns and cities, over the first 14 months of the pandemic. It takes, as its stating point, the findings of *The Doreen Lawrence Review* (2020), which explore the disproportionate impact of the current pandemic on BAME groups. We show that initially it was Black groups that were most exposed to the virus but, over the longer period, the Pakistani group has had the highest exposure. Differences between the ethnic groups reflect but are not fully explained by the regional geography of where the disease has been most prevalent over the study period.

The early exposure of Black and, especially, the Black African group is starkly evident in mortality statistics from the Office for National Statistics (ONS, 2020c). These show that the rate of death involving coronavirus among Black African males was 3.8 times higher than for those of White background. For Black African females it was 2.9 times higher. All groups other than the Chinese had a statistically significant higher rate of mortality than those of a White ethnicity. However, those ONS data are for deaths only up to 28 July, 2020, covering the first wave of the disease. Similarly, *The Doreen Lawrence Review*, is informed by data from the first half of that year. The timing of the data matters because there have been regional and sub-regional geographies to the disease that have changed as the disease has spread, contracted and spread again across the UK. The shifting geography is why, at various stages of the pandemic, some places have faced greater constraints on people mixing and on businesses trading—the ‘local lockdowns’ and tiered system of place-based restrictions that were operated to try and contain the disease.

The spatio-temporal variations in the disease would be of less relevance if the prevalence of the various BAME groups was random or uniform across the country but it is not. Consequently, the demographic characteristics of the at-risk population are changing with the geography of the disease. At the beginning of the pandemic, cases were disproportionately found in London, a geographical attribute that goes some way to explaining the higher impact upon Black communities in those early months (Harris, 2020). Later on, it was more greatly affecting Asian groups in places such as Leicester.

This paper looks at neighbourhood levels of infection over the period from the seven days ending 21 March 2020, to the seven ending 15 May 2021. It is a period over which the initial wave of the pandemic rose, spread and then contracted into more regionalised clusters of infection during the spring and summer of 2020, spreading nationally again in a second, autumn and winter wave that continued into the spring of 2021. The second led to more infections than the first, driven by a more infectious, mutated strain of the virus that emerged towards the end of 2020.

A Bayesian Poisson model is developed to identify, for each week of the study, the neighbourhoods in English major towns and cities that have the highest rates of infection. The model allows neighbourhoods to be identified that have the highest prevalence of the disease relative to the national rate and, additionally, to their immediate context. The question is then one of who lives in those neighbourhoods with higher case numbers: is it disproportionately members of one or more BAME groups? If so, do those inequalities in exposure persist or change over time? To answer, an index of exposure is developed, based on the type found in studies of ethnic segregation but here measuring the relative exposure of each ethnic group to neighbourhoods of COVID infections.

In writing this paper, we are conscious of the criticism that the BAME initialism has attracted recently, including in the report by the Commission on Race and Ethnic Disparities (2021), *The Sewell Report*, which calls for it to be dropped. That Commission regards BAME to be opaque, hiding the diverse experiences of different ethno-cultural groups that neither have all the same outcomes in, say, health or education, nor, in the framing of the Commission’s research, necessarily do worse than the majority, White British group (which is itself highly heterogenous).

We agree with this concern, noting that to use BAME as an abbreviation/synonym for ethnic minority groups (plural) need not and should not entail treating those groups as a single entity. Indeed, an important part of this paper is to reveal the differences, showing that exposure to COVID-infected neighbourhoods is unevenly distributed across BAME groups in England’s major towns and cities. It is especially the Pakistani but also the Bangladeshi and Indian groups that have had the highest exposures for most weeks. Those higher values, for the Pakistani group, remain evident when the index is stratified into deciles based on local property prices, suggesting they are not simply a function of neighbourhood deprivation. Environmental exposure, measured as residential density (people per dwelling), appears to be an important exploratory factor, with occupational exposure also contributing to the risk. However, having allowed for these factors, differences between the towns and cities, and between the groups remain.

## Literature review and context

The *Doreen Lawrence Review* (2020) was published by the UK Labour Party as a response to the COVID-19 pandemic in England, looking at structural inequalities in who is infected and dying from the disease. Its foreword argues that “Black, Asian and minority ethnic people have been overexposed, under protected, stigmatised and overlooked during this pandemic.” At about the same time, a report by the Institute for Public Policy Research (IPPR) and Runnymede Trust described the extra risk of death in minority ethnic groups as “one of the starkest health inequalities in recent times” (Patel et al., 2020). It estimated that “over 58,000 and 35,000 additional deaths from COVID-19 would have occurred if the white population had experienced the same risk of death from COVID-19 as the black and south Asian populations respectively [over the period between March and May 2020].” Those numbers would nearly double or more the actual 38,122 deaths that occurred. At the end of November 2020, Channel 4, one of the five main terrestrial channels in the UK, broadcast a documentary, entitled ‘*Is COVID Racist?*’, picking-up on occupational exposure in the medical profession and asking, “why so many Black, Asian and Minority Ethnic NHS colleagues have died from COVID-19” (Channel 4, n.d.).

The impression that BAME groups are at greater risk is supported by the data. Although published by a political party, those found in *The Doreen Lawrence Review* are not partisan but are taken from or support the findings of other studies, primarily from the first half of 2020. Early analysis, published by Public Health England in June 2020, showed that Black ethnic groups were, at that time, most likely to be diagnosed with COVID-19 and that death rates were highest among Black and Asian groups. It observed that “this is the opposite of what is seen in previous years, when the mortality rates were lower in Asian and Black ethnic groups than White ethnic groups” (Public Heath England, 2020, p. 6).^1^

That analysis covers the first wave of the disease in England. The first diagnosed deaths due to COVID were at the end of February/early March 2020, with the daily deaths in that wave peaking on April 21, with 1172 deaths. Sadly, that number was exceeded for much of January 2021.^2^ At first, London had the highest death rate of any English region. This is reflected in the higher rates for, especially, Black but also Asian groups, at that time, because these groups are disproportionately resident in the capital. According to the most recent Census for which data are presently available, 59 per cent of the entire Black residential population of England lived in London (on 27 March, 2011) and 36 per cent of the Asian population. In comparison, 15 per cent of the English population were London residents. However, it is not simply a ‘London effect’. If it was, it would apply equally to all ethnic groups who live there whereas, within London, areas with higher percentages of White British, ‘White Other’ and Chinese populations tended to have lower death rates, and those with more of the Black, Bangladeshi, Indian and Pakistani groups tended to have higher (Harris, 2020).

Those between-group differences were an early indication of what is later stressed in a report by the UK Government Equalities Office and Race Disparities Review (2021): “that ethnic minorities should not be considered a single group that faces similar risk factors in relation to COVID-19. *Different ethnic groups have experienced different outcomes during both waves of the virus*” (emphasis added). Some of those differences are at a regional scale, becoming increasingly evident through the summer and early autumn of 2020 in Government responses to the pandemic, as attention focused on towns and cities in the Midlands, North East and North West of England—regions within which local lockdown regimes were put in place to curb rising infections after the first national lockdown ended.

In this later period, ethnic inequalities still persisted but shifted more to Asian groups. When the period 20–26 August 2020 is compared with 28 May to 3 June, the number of tests had increased by 83 per cent for Asian/Asian British groups, by 44 per cent for White groups but by only 28 per cent for Black/Black British groups (Department of Health & Social Care, 2020, Table 2). This reflects the spread of COVID-19 outside of London, in places such as Oldham, Bradford, Blackburn, Manchester, Rochdale, Northampton and Leicester, where there are more of the Asian groups. Analysis by the Office for National Statistics (ONS, 2020b, Fig. 8) showed that COVID-19 had a proportionally higher impact on the age-standardised mortality rates in the most deprived areas of England throughout the period March to July, 2020, many of which are occupied by BAME groups (but not only BAME groups) and many of which are outside of London, the South East and the South West.

Attention returned to London prior to Christmas 2020 when a more transmissible strain of the virus arose whilst the city was in one of the less restrictive COVID tiers—restaurants and gyms were still open, for example—quickly leading to a spike in cases in and around the capital and, by the first week of January 2021, a further national lockdown. The easing of that lockdown began in March 2021, continuing into April when ‘non-essential’ shops and services could re-open. As of 19 May 2021, the English rate of infections is 21.2 per 100,000 population for the preceding 7 days but still over 100 in Bolton (in the North West region, with a rate of 283.1), Bedford (in the East region, 122.9) and Blackburn (North West, 114.9).

What explains these geographical variations? In England, one study identified the main predictors of COVID-19 vulnerability to be the proportions of the population, (a) living in care homes, (b) admitted to hospital in the past five years for a long-term health condition, (c) from an ethnic minority background, and (d) living in overcrowded housing (Daras et al., 2020). The co-linearity of these variables with each other, as well as with age and occupation, could explain why income deprivation was found to be statistically insignificant; that is, the effects of income deprivation were measured through the other variables.

However, it has been argued that the “inequalities in outcomes for different ethnic groups are driven by risk of infection, as opposed to ethnicity alone being a risk factor” (Commission on Race & Ethnic Disparities, 2021, p.221, after Government Equalities Office/Race Disparity Unit, 2021). In other words, it is not ethnicity, per se, that generates different rates of infection and of mortality but differences in who and what those groups are exposed to as they live and work. Occupations with increased risk of exposure to COVID include frontline medical staff, the emergency services, public transit workers, teachers and those working in the hospitality industry, many of which—for example, pharmacists, dental and medical practitioners, and bus drivers—have a disproportionate percentage of their workforce from BAME backgrounds (ONS, 2020e). Arguing that ethnicity is not the cause should not distract attention from the consequence: some groups are more affected than others.

In the United States, the Centers for Disease Control and Prevention observe that “[s]ome of the many inequities in social determinants of health that put racial and ethnic minority groups at increased risk of getting sick and dying from COVID-19” include discrimination, healthcare access and utilisation, occupation, educational, income and wealth gaps, and housing (CPD, 2020). These echo the concerns found in *The Doreen Lawrence Review*. A systematic review of 50 studies, 42 from the United States of America and 8 from the United Kingdom, confirms that individuals from Black and Asian ethnicities had a higher risk of COVID-19 infection compared to White individuals (Sze et al., 2020) although, as we shall see, that does not always remain true when focusing specifically on urban areas in England and omitting those that are more rural.

## Analytical approach

### About the infections data

The main dataset that is used in this paper is from the official UK Government data dashboard (UK Government, n.d.). Here we focus on cases of COVID-19 infection rather than deaths because only the infection data have been regularly available at weekly intervals and at a neighbourhood scale in England, allowing geographical and temporal patterns to be considered.^3^

The neighbourhood geography is the Middle Level Super Output Areas (MSOAs). These are the third tier of the Census geography for England and Wales; third when aggregating upwards from the smallest, which are Output Areas. They represent a formal, administrative specification of neighbourhood but they are not arbitrary. They were designed with the criteria of broadly equal population size, socio-economic homogeneity based on accommodation type and tenure, and spatial compactness of the zones (Cockings et al., 2011). Matching them to the Office for National Statistics mid-2019 population estimates suggests an average population size of 8605 in English major towns and cities, with an interquartile range from 7154 to 9640 persons.

The infections data tally, by date reported, the number of individuals who had at least one positive COVID-19 test result, over that and the preceding six days. They span the 61 weeks from the seven days to 21 March 2020 to the seven days to 15 May 2021. The numbers are underestimates of the true prevalence of the disease within the population because not everyone who has the disease is tested or displays symptoms. The data include only pillar 1 cases until 2 July, from when pillar 2 cases also are included. Pillar 1 cases are “diagnostic tests done in Public Health England labs and hospitals for health and care workers and patients who are seriously ill” (Full Fact 2020). Pillar 2 cases “are also diagnostic tests but done by commercial partners for the wider population. These tests are done at regional test sites, mobile testing units, satellite test centres and via home tests.” Even after the inclusion of pillar 2 data, on-going problems with the track-and-trace system meant about half of those who had been in contact with an infected person had not been contacted (Triggle et al., 2020) so could harbour the disease without knowledge.

The undercount is compounded by suppression of the exact number of cases in any MSOAs that had between 0 and 2 cases each week. For this study, these are treated as zero values but they could be one or two. In principle, it would be possible to aggregate the values to the local authority scale and compare the totals with the published number of cases for each authority. The differences are the total number of suppressed cases per authority, which could then be allocated to the ‘zero’ MSOAs. A problem with doing-so is that it will generate non-integer values that would need to be rounded up or down for the Poisson modelling. The more fundamental restriction is that the higher-level geography used for the analysis—that of English major towns and cities—does not fully match with local authorities or any other geography for which the non-censored COVID-19 counts are published. It would still be possible to establish the number of undercounted cases but not where those infected people live, which could be outside the towns and cities. Our reason for using this urban geography is explained in the following section.

The analysis assumes that tests follow symptoms, as well as the identification of known clusters of the disease and, therefore, that the data sufficiently well track the disease’s spread amongst the population to be broadly representative of who has been infected and where they live. However, they are neither a random sample nor a complete enumeration of all those who have been infected each week. Prior to July they are, in effect, a count of the number hospitalised by COVID-19. From then on, they are a function of testing—of where people are being tested and their ability to access a test. That testing capacity has been constrained for some of the period of this study, with the British Medical Journal publishing a briefing in September entitled *What’s going wrong with testing in the UK?* (Wise, 2020). The 7-day average numbers of tests at the beginning of the months from May 2020 to May 2021 were 67,512 (May 2020), 92,716 (June), 112,001 (July), 154,151 (August), 187,287 (September), 255,302 (October), 284,306 (November), 311,602 (December), 425,064 (January), 644,440 (February), 682,194 (March), 808,946 (April) and 485,640 (May 2021) (UK Government, n.d.). Although the increase in the first third of 2021 is a response to the second wave of the disease, it also reflects increased capacity to test, which, in turn, allows for more positive diagnoses. The drop, in May, comes as the number of cases were at the lowest for the year. Cases rose again the following month.

Despite their deficiencies, the data are the official source of infection data in the UK, used to inform public health policy and to guide preventative measures such as national and local lockdowns. The most likely systematic bias is an undercount of those who might be infected but can least afford either to socially isolate from out-of-home employment for the quarantine period if tested positive or to travel to access a test. If such a bias exists, then it is likely to downplay the infection rate amongst Black, Pakistani and Bangladeshi groups because of the intersections of ethnicity and social disadvantage (Commission on Race & Ethnic Disparities, 2021). This will lead to more conservative estimates of the differences in exposure between them and other groups.

Finally, and whilst our focus in on infections, at the end of February 2021, a data set giving the number of monthly deaths due to COVID per MSOA was published, having not previously been updated since August 2020 (ONS, 2021b). It was further revised at monthly intervals, giving a span, at the time of writing, from March 2020 to April 2021. We use these data to consider whether what is seen in the infection data is evident in the mortality data too albeit with less temporal granularity.

### Geography of the study

The study is confined to English major towns and cities, as defined in the ONS file of December 2015 (ONS, n.d.). This is based on the built-up areas geography developed following the 2011 Census (ONS, 2013). Rural and semi-urban areas are not considered, partly because of their greater population sparsity that, all things being equal, reduces person-to-person contact and therefore the transmission of COVID-19. Much of the South West region of England, for example, has had consistently lower rates of infection throughout the pandemic and is mainly rural (see Fig. 3, below).

However, the main reason is demographic, informed by the ethnic geography of the country. Whereas many English towns and cities are ethnically diverse, more rural areas typically are not, containing far fewer of the BAME than White populations. According to the 2011 Census, 61 per cent of the White British population resides outside the major towns and cities. The next largest percentage is equal for the White Other and Mixed ethnicity groups, of whom 30 per cent do not reside in these towns and cities. For the Black Caribbean and Black African groups, it is only 11 per cent. Nationally, lower COVID-19 infection and death rates amongst the White British population reflect rural–urban patterns of living. To compare ‘white’ rural hamlets with multicultural urban settlements is problematic; they are very different types of places. Consequently, we prefer the direct comparison, asking whether differential rates of exposure remain evident between different ethnic groups, within urban settlements. This decision means that the White British exposure to COVID-19 infected neighbourhoods is over-stated relative to what would result if rural areas were included too.

The ONS definition of the towns and cities avoids the artificial truncation of, for example, Bristol’s urban boundary at the border with South Gloucestershire. The general problem is that the built-up extent of some towns and cities is not congruous with the administrative areas (local authorities) within which they fall or span across. The built-up area geography aims to provide a more geographically exact definition of the major towns and cities, although being based on 2011 Census data, they are likely to be underbounded, on the basis that most will have continued to expand outwards over the last decade.

In total, 109 English towns and cities are included in the study. The smallest is Walsall, with a mid-2019 estimated population of 65,928. The largest is London, with a population of 8,924,265. Because London is so much larger than the other settlements—its population is over 35 times greater than the average of 250,479 and approaching eight times greater than the second largest settlement, Birmingham, with 1,153,804—it is split into its 32 local authorities for the analysis: the 32 London Boroughs, with the City of London merged with Westminster. This gives a total of 140 urban places.

### Method of estimating the relative exposure of ethnic groups to neighbourhoods of COVID-19 infection

For the analysis, an index of exposure is formed to measure how much members of the various ethnic groups are exposed, by residence, to neighbourhoods of COVID-19 infection for each week of the study period. This index is based on the residuals from a Bayesian Poisson model, which are used to identify neighbourhoods that have more or less than the expected number of COVID-19 cases, relative to: (a) the overall infection rate that week for all the towns and cities plus some control variables; or, (b), that overall rate, the control variables and also relative to the town or city in which the neighbourhood is situated.

The underlying model is,

$$y_{i } \sim {\text{Poisson}}\left( {\lambda_{i} } \right)$$

where

1 $${\text{log}}\left( {\lambda_{i} } \right) = { }\beta_{0} + \beta_{1} x_{1} + \beta_{2} x_{2} + \beta_{3} x_{3} + {\text{log}}\left( {P_{i} } \right)$$

here $y_{i }$ is the expected number of infection counts in MSOA, $i$, conditional on the control variables ($x_{1}$, $x_{2}$ and $x_{3}$,). The control variables are standardised into *z-*values in the model (units of standard deviation from their mean). These control variables are the ratio of care home beds in the MSOA to the adult population, the percentage of the adult population aged 18 to 21, and the percentage aged 22 to 35. The first two of these variables are included to control for two distinct types of setting where infection rates have been unusually high for at least some of the pandemic but where the inhabitants of those settings do not necessarily reflect the population characteristics of the wider neighbourhood. These are care homes and student halls of residence.

The third variable, the percentage of the adult population aged 22 to 35, is included because of a period, after the first national lockdown, when infection cases were growing amongst younger adults, and because BAME groups are, on average, younger than the White British. There is a risk that this control ends-up understating the exposure of BAME groups to infected neighbourhoods if controlling for age also partially controls for ethnicity. However, we judge this preferable to potentially confusing an age effect with an ethnic one. From an analytical point of view, if observed differences remain between the ethnic groups despite potentially biasing those differences downwards then that strengthens the argument that those differences are of substantive interest.

No further variables are included in the model because its purpose, at this stage, is not to explain the variations in the infection rates but to reveal them, at the neighbourhood and town/city/London Borough scales. It is adopted to enable a multilevel approach that permits the differences *between* the urban places to be separated from the differences *within* them. It is essentially a null model—a model that partitions, between the levels of the model, the variance around the average of the response variable, without seeking to explain that variance statistically. It is not quite that because of the control variables that allow for a few distinct circumstances that are unusual and would otherwise inflate the more typical number of cases in their MSOA. But it is a baseline model, used to reveal places of high or low infection and, by extension, who is exposed to them. Looking for the causes of the variation is largely left as to later in the paper on the basis of measuring first then explaining.

Returning to Eq. (1),$P_{i}$ is the number of the adult population in the MSOA and ${\text{log}}\left( {P_{i} } \right)$ is an offset, with a prescribed coefficient of one. These give the log of the conditional rate of infection, seen by re-arranging the equation,

$${\text{log}}\left( {\lambda_{i} } \right) - {\text{log}}\left( {P_{i} } \right) = { }\beta_{0} + \beta_{1} x_{1} + \beta_{2} x_{2} + \beta_{3} x_{3}$$

$${\text{log}}\left( {\frac{{\lambda_{i} }}{{P_{i} }}} \right) = { }\beta_{0} + \beta_{1} x_{1} + \beta_{2} x_{2} + \beta_{3} x_{3}$$

The multilevel model is formed by extending the underlying model to include two sets of random intercept terms, allowing for extra-Poisson variation at the MSOA level and for differences between the towns/cities/London Boroughs:

2 $$\begin{gathered} {\text{log}}\left( {\lambda_{i} } \right) = { }\beta_{0} + \beta_{1} x_{1} + \beta_{2} x_{2} + \beta_{3} x_{3} + \log \left( {P_{i} } \right) + \varepsilon_{i} + \delta_{ij} \hfill \\ \varepsilon_{i} \sim {\text{Normal}}\left( {0,\sigma_{\varepsilon }^{2} } \right) \hfill \\ \delta_{ij} \sim {\text{Normal}}\left( {0,\sigma_{\delta }^{2} } \right) \hfill \\ \end{gathered}$$

The two parameters, $\varepsilon_{i}$ and $\delta_{ij}$, measure the deviation from the expected log infection rate at the MSOA and place levels. They measure the within and between place differences, respectively. It is these that are used to form the index of exposure, measuring how exposed the average member of each ethnic group is to neighbourhoods with higher or lower than the expected infection rate.

The index is based on that used in studies of segregation where,

3 $$I_{k} = \mathop \sum \limits_{i = 1}^{N} \frac{{n_{i\left( k \right)} }}{{n_{ + \left( k \right)} }} p_{i}$$

For such studies, $I_{k}$ is the index value for ethnic group, $k$, of which there are $n_{i\left( k \right)}$ in neighbourhood, $i$, and $n_{ + \left( k \right)}$ for all $N$ neighbourhoods within the study. The remaining value, $p_{i}$, may be interpreted probabilistically. For a segregation index, it is the probability of selecting a member of a second ethnic group, $l$, from the same neighbourhood that the member of ethnic group $k$ is living in: it is $p_{i} = n_{i\left( l \right)} /n_{i + }$ where $n_{i + }$ is the total population of $i$ (see, *inter alia*, the appendix of Harris & Johnston, 2020, or Kaplan, 2018).

The modification we make is $p_{i}$ becomes the probability of selecting, from a Normal distribution, a value lower than the MSOA’s deviance from its expected infection rate for the week. If most of an ethnic group is living in MSOAs where the infection rate is higher than expected then the index will tend towards one, getting closer the greater the deviance from expectation. If most are living in neighbourhoods where the infection rate is lower than expected, it will tend towards zero. If the group is spread amongst neighbourhoods where those with higher infection rates balance out those with lower (and assuming the model’s assumption of Normality of the random intercepts holds) then the index value will be 0.5.

Specifically, $p_{i}$ is extracted from $\varepsilon_{i}$ and $\delta_{ij}$ in Eq. (2). These are treated as quantiles from a Standard Normal distribution and used to generate the probabilities that feed into the index of exposure. With reference to Eq. (3), $p_{i} = P\left( {z < \varepsilon_{i} + \delta_{ij} } \right)$ or $p_{i} = P\left( {z < \varepsilon_{i} } \right)$ where $z$ represents quantiles from a Standard Normal distribution. Critically, the two variants of $p_{i}$ mean that the index can be calculated with or without place effects, the latter controlling for the differences between towns and cities and therefore the broader scale geography of the disease. This is discussed further in the results section.

The model is fitted 61 times, once for each week of the pandemic included in the study. It follows that the index of exposure is calculated weekly too. Adding-in the subscript, $t$, to denote time gives,

4 $$\log \left( {\lambda_{it} } \right) = \beta_{0t} + \beta_{1t} x_{1} + \beta_{2t} x_{2} + \beta_{3t} x_{3} + \log \left( {P_{i} } \right) + \varepsilon_{it} + \delta_{ijt}$$

This shows that the modelled rate varies weekly as do the random intercepts but not the control variables because the population size, demographic profile and number of care homes beds are taken to be constant for the study period. The coefficients attached to these control variables are permitted to vary because there is no reason to assume that any of the controls is of equal importance across all weeks. In fact, the number of care home beds per adult population had greatest association with increased neighbourhood infection rates as the first national lockdown ended, in May 2020, when concern about the lack of protection afforded to care home residents and their staff was of considerable media interest (Calvert & Arbuthnott, 2021). Greater percentages of those aged 18–21 generally has a negative association with infection rates but not in the first week of October when it had the strongest association with increasing those rates. Coincidentally or otherwise, this is when students returned from their homes to their term-time addresses after a delay in the start of the University term. The association of populations aged 22–35 with raised infections was greatest through the summer of 2020 when pubs and restaurants had partially reopened and more people had returned to their workplaces, and again towards the end of the study period, in April and May 2021 when the third lockdown had been eased.

The models are fitted as a Bayesian model using the brms package for R (Bürkner, 2017, 2018), which provides an interface to Stan (Stan Development Team, 2019). Results takes the form of draws from a Bayesian posterior distribution for the parameters of interest that are then summarised by their mean value. The data and a tutorial in how to replicate the models are available at https://rpubs.com/profrichharris/ASAP.

## Results

**Fig. 1:**
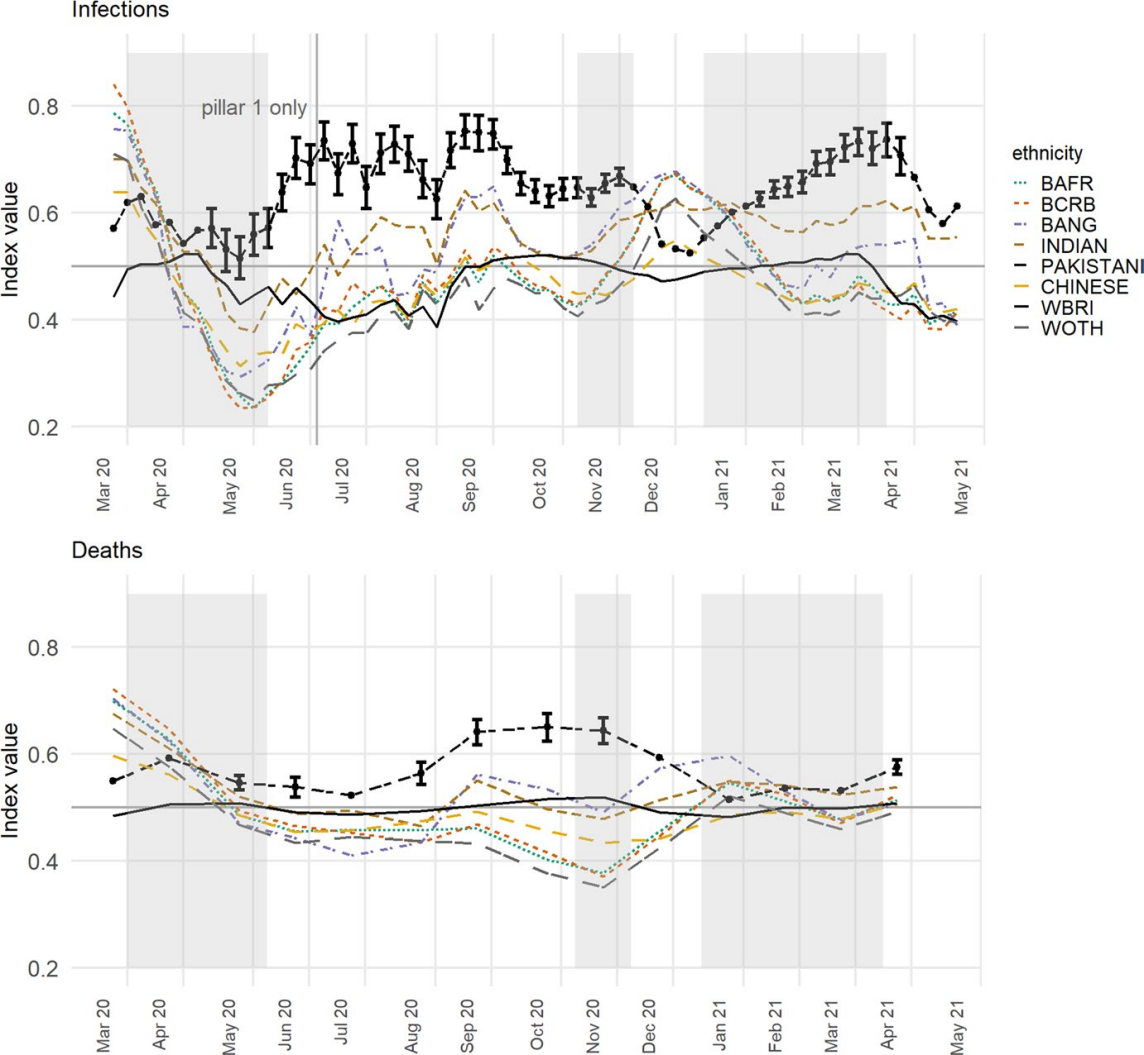
Showing (upper) the Index of Exposure of the various ethnic group to COVID-infected MSOAs for each week of the study, and (lower) recalculated for mortality rates. The ethnic groups are *BAFR* Black African, *BCRB* Black Caribbean, *BANG* Bangladeshi, *WBRI* White British and *WOTH* White Others. Prior to July 2 only ‘pillar 1’ tests are included in the infection data. The English national lockdown periods are shaded in grey. Observations highlighted with an error bar are the ones where the lower bound of the 95% bootstrapped interval for the Pakistani group does not overlap with the upper bound for any other group. (See text for details)

The index values, initially calculated with the inclusion of both the MOSA and place level effects, $\varepsilon_{i} + \delta_{ij}$, are shown in the upper part of Fig. 1. Because the model is fitted separately for each week, the values are relative: they show which of the ethnic groups that week are living more greatly or less in COVID-infected neighbourhoods, given the underlying infection rate.

To improve the legibility of the chart, some groups are omitted; specifically, the Arab, Black Other, Irish, Roma/Travellers, Other Asian, Other and joint ethnicity groups. They are omitted throughout the analysis. The Black African group, labelled BAFR in Fig. 1, formed 1.8 per cent of the residential population of England in the 2011 Census; the Black Caribbean group (BCRB), 1.1 per cent; the Bangladeshi (BANG), 0.8 per cent; Indian (INDIAN), 2.6 per cent; Pakistani (PAKISTANI), 2.1 per cent; Chinese (CHINESE), 0.7 per cent; White British (WBRI), 85.4 per cent; and White Other (WOTH), 4.6 per cent. Amongst those omitted, the Irish (1.0%), Other Asian (1.5%), Other (1.0%) and joint ethnicity (2.3%) groups formed a greater percentage of the Census population than some of those included but are removed either because they are less ‘distinct’ as a group or because they are not typically associated with BAME groups.

Figure 1 reveals that it was especially the Black groups that were most exposed to higher COVID-infected residential neighbourhoods earlier in the pandemic. This was during the period of pillar 1 testing and indicates that these groups were hospitalised more frequently than others in the first month. However, their index values subsequently decline, and steeply so, only rising again between the second and third lockdowns with the spread of the mutated virus in London and the South East. The implication is that when COVID-19 is spreading across the capital, it is the Black population that is most impacted but, at other times, the group that is most consistently over-exposed is the Pakistanis. This shift may be a function of the increased testing. When comparing 28 May to 3 June with 20 August to 26 August, the percentage increase in testing is much greater for Asians/Asian British, at 83 per cent, than it is for Blacks/Black British, at 28 per cent (Department of Health & Social Care, 2020: Table 2). However, this need not mean that the Black groups have been under-sampled if that change in testing reflects a change in the disease’s geography, which is likely.

Of interest is how often the index is highest for the Pakistani group: it is for 50 of the 61 weeks (82%). The Indian and Bangladeshi groups often have higher index values too but usually not to the same extent. To add weight to this finding, the index values were recalculated 2000 times for each of the ethnicity groups in turn and for each week, adding stochastic variation by bootstrapping from the set of deviance values ($\varepsilon_{i} + \delta_{ij} )$ with probability of selection equal to $n_{i\left( k \right)} /n_{ + \left( k \right)}$, then finding the interval between the upper and lower bounds of the ‘middle’ 95 per cent of the 2000 index values generated for each group. For 41 of the 61 weeks (67%), the lower value for the Pakistani group is greater than the higher values for all the other groups. Those weeks are highlighted in Fig. 1 with an error bar (no others are included to avoid cluttering the chart).

Similar trends are found if the index is recalculated modelling the numbers of COVID-19 deaths instead of infections. The results are in the lower part of Fig. 1. The two sets of indices are not fully comparable, partly because the mortality data are monthly instead of weekly but more especially because the mortality index is modelled with the same care homes variable used for infections but also with the percentages of the adult population aged 25 to 29, 30 to 34, 35 to 39, and so forth, to those aged 80 years or greater. The reason for including the additional age variables for mortality but not infections is that age does not cause infection—not directly—but it is the greatest risk factor for death having been infected, especially for older populations. The additional co-variates act to age standardise the mortality data. Despite the model differences, it is again the Pakistani group with most frequently the highest index value, for 10 of the 14 months (71%), of which 7 are distinct from the rest using the bootstrapped intervals.

Both sets of results lend support to the Commission on Race and Ethnic Disparities’ (2021) view that ethnic minorities are not a homogenous group that always all are at the lower end of social or, in this case, health inequalities. There appear to be many occasions when the average Chinese or Black person, for example, is living in an urban neighbourhood with fewer infections than the average White British one. We acknowledge that the ethnicity data underpinning Fig. 1 are almost a decade old and that the population as was, at the time of the 2011 Census, will neither have remained in situ in each MSOA nor have been replaced on an exact like-with-like basis as people change their place of residence. It is known, from the segregation literature, that there has been a process of desegregation in most UK towns and cities as ‘minority’ groups have moved out from their traditional enclaves into what are becoming more mixed neighbourhoods (Catney, 2015; Catney et al., 2020; Johnston et al., 2013). If that process has continued, which is likely (Harris & Johnston, 2020), and if, therefore, the Census data now overstate the geographical clustering of particular ethnic groups into particular neighbourhoods then the indices of exposure to COVID-19 could exaggerate the differences between the groups. This is something we can review when the 2021 Census data are published, between 2022 and 2023 (ONS, 2021a). Any updates will be posted to https://rpubs.com/profrichharris/ASAP.

Presently, there are few alternative sources of geographically detailed ethnicity data. Ethnic population projections are useful (Wohland et al., n.d.) but not available for the geographical zones we are using. National pupil data are more geographically detailed and updated annually but only include those of school age, in state schools (Harris & Johnston, 2020). Consumer data hold promise (Lan et al., 2020) but raise questions about their representativeness of the population and the biases they contain. In any case, it is highly unlikely that the members of the various ethnic groups have become sufficiently dispersed over the last nine years for the trends shown in Fig. 1 to be purely an artifact of dated information. For the groups to become that spread out would require the disappearance of the socio-spatial processes and spatial inequalities that led some groups to be more concentrated in some places than others—an unlikely proposition that is refuted by the evidence (Byrne, 2020).

The greater problem in interpreting Fig. 1 is not the age of the ethnicity data but the changing regional geography of the disease over the study period. The higher exposure of Black groups in the initial period of the first wave is a function of what was, at that time, the higher prevalence of COVID-19 in London, where a greater share of the Black groups lives. The subsequent decline in their exposure, superseded by Asian groups, reflects the subsequent outbreaks in parts of the Midlands and more northerly regions.

**Fig. 2:**
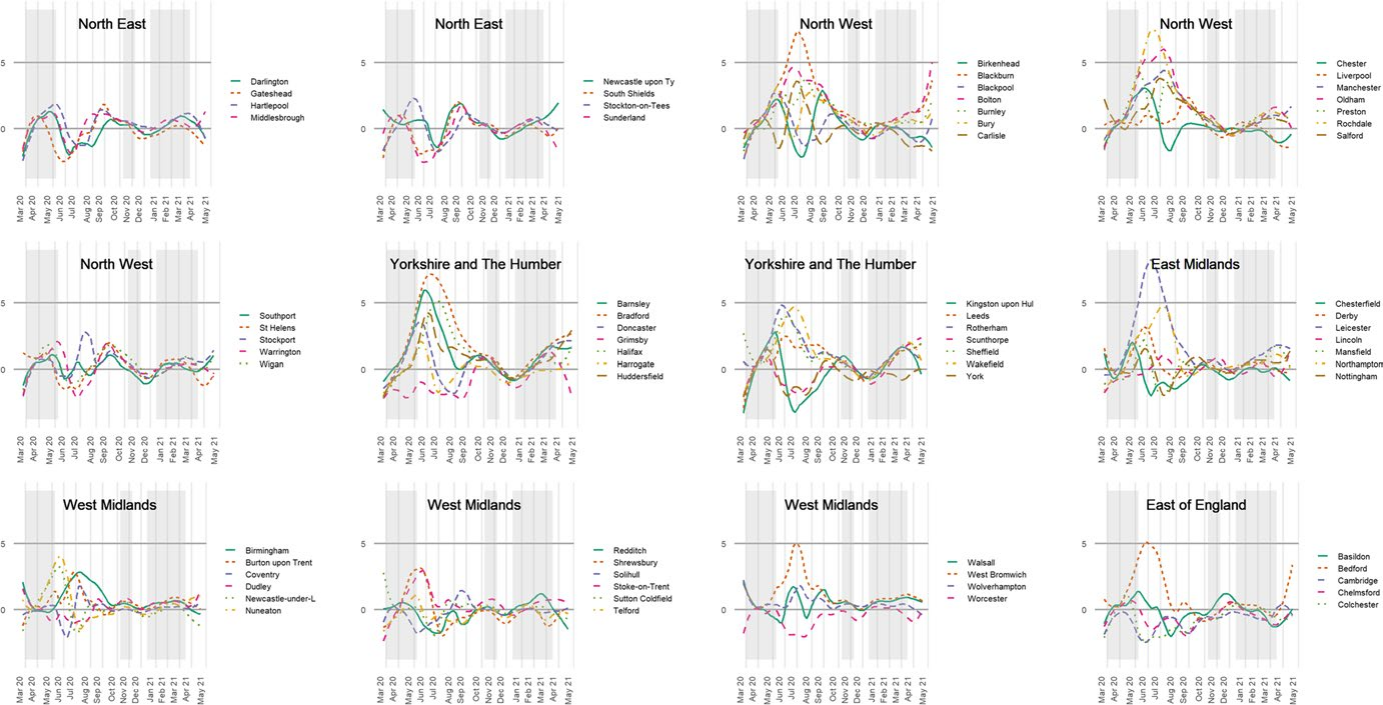

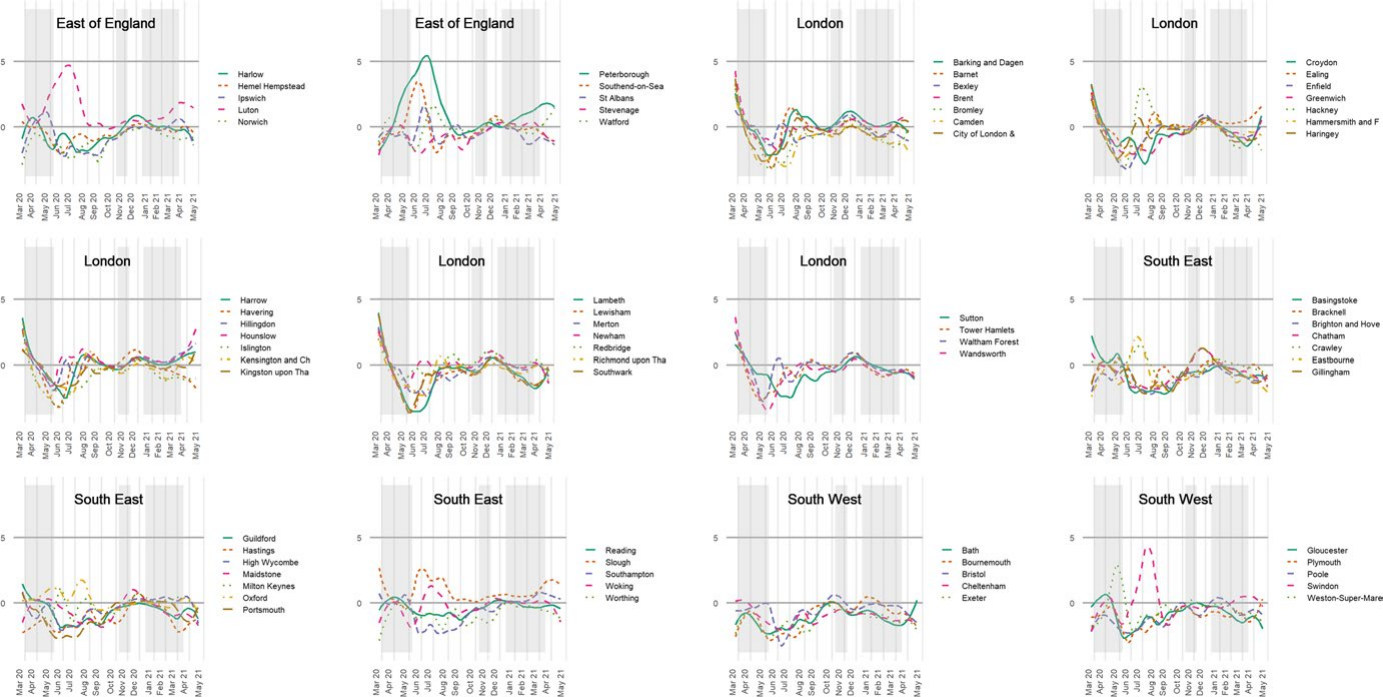
Indicating how much individual towns and cities in the English regions deviate from their expected log infection rate each week of the study. Notes: Smoothing has been applied and the following names have been truncated: Barking and Dagenham, City of London & Westminster, Hammersmith and Fulham, Kensington and Chelsea, Kingston upon Hull, Kingston upon Thames, Newcastle-under-Lyme, Newcastle upon Tyne, and Richmond upon Thames

This changing geography of infections is revealed by plotting $\delta_{ij}$, the deviance from the expected log infection rate for that week at the town and city level. The plots are in Fig. 2, to which some smoothing has been applied to improve the visual clarity of the charts, drawing out the broad trends.^4^ It confirms that London had higher than expected rates early-on but those quickly tailed off. In the middle period, between the first two national lockdowns, the infection rates were higher than expected in parts of the North West, Yorkshire and The Humber, and in the East Midlands—in places such as Blackburn (about 27 per cent Indian, Pakistani or Bangladeshi in its 2011 Census population), Rochdale (13%), Liverpool (2%), Oldham (18%), Bradford (25%) and Leicester (32%). Most of these have comparable or greater percentages of the three Asian groups than London (12%), except for Liverpool. By the first months of 2021, most places had converged around the zero line in Fig. 2. This is not because the number of infections was reducing. Quite the opposite; it was because the disease had spread so widely that there was less variation between places. Some differences have re-emerged as the most recent lockdown restrictions have been eased.

These geographical variations are not unimportant but invite the question of how they arose. Why did Leicester, for instance, have a period of greater infections necessitating a local lockdown? The answer is likely to lie within its borders—the housing stock, for example, or employment types, amongst other attributes of the city and its population. It has been suggested that Londoners’ initially higher exposure to COVID-19 was because it is a world city with greater travel connections both to the rest of the UK and across the world, as well as within London itself, with a much more extensive public transport system than other English towns and cities (Harris, 2020). In seeking to understand the geography of the disease, broad contextual differences between places matter. To discount them could downplay the likelihood that what occurred in Leicester, London or wherever was not purely happenstance but reflects some general characteristics of those cities, increasing the exposure of their populations to COVID.

Nevertheless, even within places where the overall infection rate is high, it is possible that some groups are living in neighbourhoods where the rate is even higher. Or, in places where the overall rate is low, still some groups face much higher exposure. It is instructive to modify the index, removing the broader scale differences between the towns and cities and leaving the exposure measured relative to the local context. If it emerges that one or more groups are still more exposed to COVID-19 than others then it cannot be attributed only to the broad, regional geography of the disease. It means that the differences between the groups also emerge within individual towns and cities, not just between them.

**Fig. 3:**
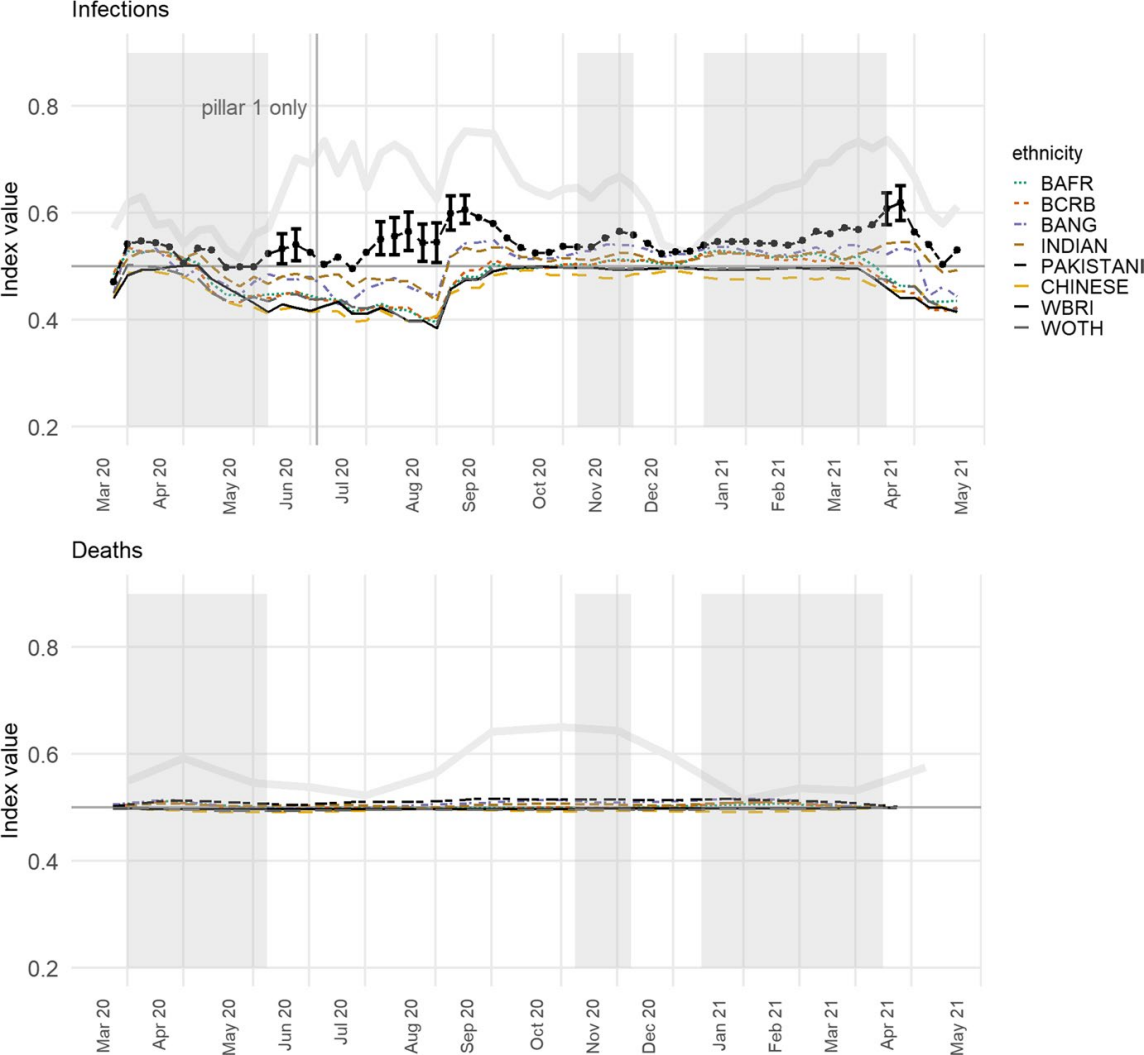
Showing the Index of Local Exposure values, which controls for the broader scale geography of the disease each week and is based on the variations within towns and cities rather than between them. The previous values for the Pakistani group are indicated in light grey

The way to make the modification is to recalculate the index of exposure without the place effects from the underlying model. In terms of Eqs. (2 and 3), above, this means using only $\varepsilon_{i}$, and not $\varepsilon_{i} + \delta_{ij}$, giving an index of local exposure. The results are shown in the upper part of Fig. 3, drawn to the same scale as Fig. 1 on the y-axis. The differences between the ethnic groups have reduced—unsurprisingly as we have removed the geographical variation between the towns and cities (but not, to re-iterate, within them)—but, in what remains, for 59 of the 61 weeks (97%), the Pakistani group had the greatest local exposure, of which 55 weeks are consecutive.

Those higher values are less distinct than they were previously. Using the bootstrapping procedure, now sampling from $\varepsilon_{i}$, the lower limit of the 95 per cent interval for the Pakistani group is greater than the upper limit for any of the other groups on 11 occasions (18%), reduced from 41. Those occasions are concentrated in the period between the first and second lockdowns, especially in the summer of 2020. As national rates of infection fell, Pakistanis were more likely to be living in neighbourhoods where rates remained locally higher. We believe that this finding should not be understated because even under a restricted scenario that focuses only on urban areas, which removes the broader-scale patterns in the disease and allows for uncertainty in the index scores, still the Pakistani group is found to have been exposed disproportionately to the higher COVID-infected neighbourhoods than are other groups. The Chinese and the White British are often the least exposed groups, at the local level.

However, the differences all but disappear between the ethnic groups when the index is recalculated for the age-standardised mortality rates. The Pakistani group does still have the highest value for 10 of the 14 months but the differences are trivial and illegible on the chart. The implication of this is that urban inequalities in mortality are more strongly expressed at the regional level and between major towns and cities (Griffith et al., 2021), whereas inequalities in infection are evident within towns and cities too.

## The effects of environmental and occupational exposure

The preceding analysis shows that some BAME groups have faced greater exposure to COVID-19 but not uniformly so and not always at the same time. Amongst them, it is the Pakistani and, to a lesser extent, the Bangladeshi and Indian groups that have had the greatest exposure to COVID-19 infected neighbourhoods within English major towns and cities. This remains true after controlling for the variations between the towns and cities and therefore for the broader scale geography of the disease.

**Fig. 4:**
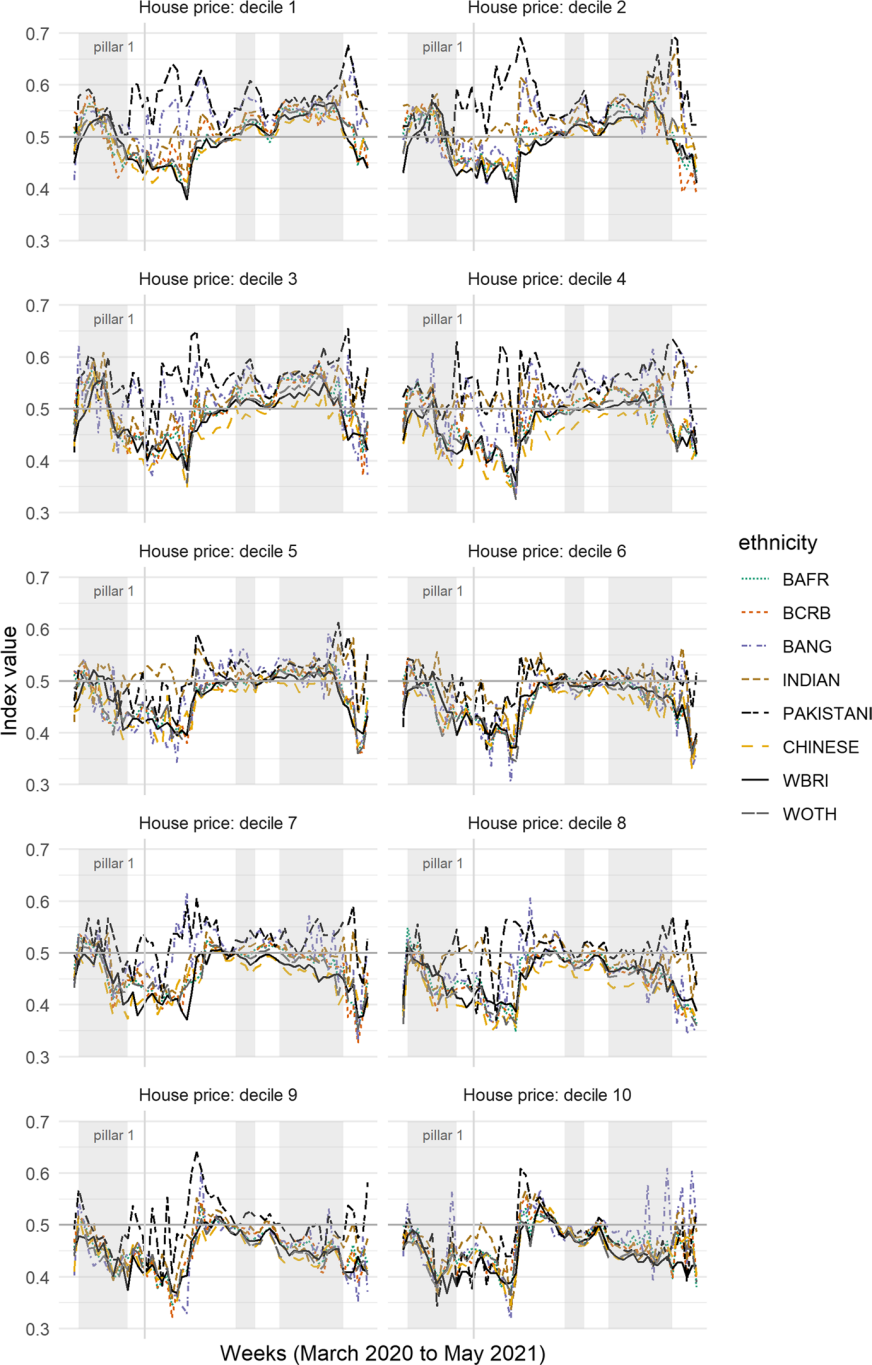
Showing the Index of Local Exposure values for each ethnic group, stratified by the average house price value of the areas in which they live. Decile 1 is the lowest average, decile 10 the highest, calculated relative to the town or city in which the properties were sold

Furthermore, the finding is not confined to what might be regarded as the most economically disadvantaged neighbourhoods because it is broadly consistent for areas of lowest and of higher average house price. To evidence this, Fig. 4 shows the results from recalculating the index of local exposure with each of the ethnic groups split into ten sub-groups according to the decile of the trimmed mean house price of the properties sold in their MSOA between January 1, 2017 and February 27, 2020. The deciles are calculated on a town and city basis, in this case treating London as a whole. This means that those living in the highest decile are in MSOAs with the most expensive property, on average, for their town and city, not necessarily nationwide. This is to prevent the top decile being dominated by London.

As before, it is not always the magnitude of the differences that are of most interest but the consistency of them disadvantaging the Pakistani group. This appears not to be a straightforward function of neighbourhood deprivation because the greater exposure of the Pakistani group to COVID-infected neighbourhoods is not restricted to the neighbourhoods of least cost housing. It is certainly present in those neighbourhoods: for decile 1, the Pakistani group has the highest index value of all groups for 50 of the 61 weeks (82%). However, that number is equalled by that for decile 9—amongst the most expensive housing—for which the index value is also highest for the Pakistani group for 50 of the weeks. Not too dissimilar values are found for deciles 3 (46 weeks), 4, 7 and 8 (each 39 weeks), and 2 (38 weeks). The number is lower for decile 6 (28 weeks) but it is only for deciles 5 and 10 that Pakistanis are not the most frequently exposed group. For these two deciles, the frequency is exceeded by the Bangladeshi and Indian groups. It is important to be mindful of the ecological fallacy. It does not follow that those Pakistanis who live in neighbourhoods with higher housing price (on average) are themselves living in property that is expensive to buy or rent. However, one way to make such property affordable is to share the space with others—a possibility we return to shortly.

It also does not follow that because their neighbourhoods have higher infection rates that it is members of the Pakistani group who are infected (because other groups live in those neighbourhoods too). However, given that COVID is a disease spread by coming into close contact with someone else who is infected, it is reasonable to argue that the risk is raised. Why, then, is the Pakistani group, especially, living in neighbourhoods with higher infection rates locally? *The Doreen Lawrence Review* identifies two key factors: environmental exposure—poor quality and overcrowded housing—and occupational exposure. Evidence for the first of these is suggested by looking at the residential density of each MSOA, calculated as the mid-2019 population estimate divided by the number of domestic mail delivery points per MSOA.^5^ For the average member of each ethnic group, the residential density is, in ascending order: 2.28 people per dwelling for the White British; 2.48 for the White Other group; 2.49 for the Chinese; 2.58 for Black Africans; 2.60 for Black Caribbeans; 2.67 for Indians; 2.72 for Bangladeshis; and, 2.79 for Pakistanis.

On average, therefore, members of the Pakistani group are living in higher residential density areas. Moreover, this result holds for nearly every one of the property price deciles: the residential density for the average Pakistani group member is greater than for any other group, except in deciles 5, 9 and 10. The densities are shown in Table 1, which also reveals that the residential density is always lowest for the average member of the White British group.

**Table 1:** The estimated residential density (people per dwelling) for the MSOA of the average member of each ethnic group, by the decile of the average house price of the MSOA in English major towns and cities

Decile	BCRB	BAFR	BCRB	BANG	Indian	Pakistani	Chinese	WBRI	WOTH
1	2.65	2.66	2.65	2.84	2.70	2.89*	2.50	2.34^#^	2.50
2	2.70	2.67	2.70	2.93	2.86	3.03*	2.54	2.37^#^	2.58
3	2.68	2.64	2.68	2.93	2.92	2.94*	2.50	2.35^#^	2.57
4	2.54	2.53	2.54	2.81	2.80	2.91*	2.54	2.33^#^	2.49
5	2.66	2.61	2.66	2.67	2.68*	2.66	2.51	2.33^#^	2.58
6	2.60	2.52	2.60	2.65	2.68	2.70*	2.50	2.36^#^	2.58
7	2.62	2.58	2.62	2.71	2.70	2.77*	2.55	2.36^#^	2.61
8	2.51	2.51	2.51	2.51	2.56*	2.55	2.45	2.34^#^	2.51
9	2.63	2.58	2.63	2.59	2.62	2.65*	2.63	2.40^#^	2.64
10	2.50	2.49	2.50	2.48	2.59	2.52	2.64*	2.38^#^	2.57

*Highest value for the decile

#Lowest value for the decile

Again, neighbourhood relationships need not describe individual circumstances: Pakistanis are not necessarily living in higher density properties. However, according to the English Housing Survey, April 2016 to March 2019, 18 per cent of Pakistani households were overcrowded, defined as a property having fewer bedrooms than it needs to avoid undesirable sharing, based on the age, sex and relationship of household members. That percentage is exceeded only for Bangladeshis, at 24 per cent. For the White British, it is 2 per cent (UK Government, 2020). ONS data from the Annual Population Survey household dataset, January to December 2018, show that 35 per cent of Pakistanis households are intergenerational households, with someone present from each of the age groups 0–19 years, 20–69 years and aged 70 or over. Again, this is only exceeded by Bangladeshi households (56%) and lowest for the White British (1.5%) (ONS, 2020c). There are cultural, as well as economic reasons why some groups live in intergenerational households.

On face value, there is not a strong relationship between residential density and how far each neighbourhood deviates, locally, from the expected, log infection rate. A simple linear regression suggests a Pearson correlation of $r = 0.08$, with $y_{i} = \varepsilon_{i}$ as the dependent variable, the density values, mean-centred for each individual town and city, as the predictor variables, and with the separate weeks handled as fixed effects. This is a low effect size under Cohen’s (1988) criteria.

However, if the model is weighted according to the share of the total Pakistani population that lives in each neighbourhood, then the correlation rises to 0.25, which is nearing a medium effect. This is greater than for any other group. The corresponding correlation when weighting by the distribution of the Bangladeshi population is 0.16; by Indians, 0.16; by Black Caribbeans, 0.09; by Black Africans, 0.08; by White Other, 0.05; by White British, 0.05; and, by Chinese, only 0.03. In short, residential density has a greater effect on exposure to COVID-19 in the neighbourhoods where the Pakistani population is living than it does for any other of the ethnic groups.

Similar analysis can be undertaken looking at the percentage of each neighbourhood’s residents in key worker jobs. Relevant data have been collated by Oxford Consultants for Social Inclusion from a 2018 Business Register and Employment Survey as part of a larger dataset (OSCI, n.d.). The overall correlations between the local variations in the log infection rates and jobs in health, transport and storage, and education are trivial at $r = 0.03$, $0.02$ and $r < 0.01$, respectively. However, weighting by ethnicity gives correlations of 0.19 for each when weighting by the Pakistani group, 0.10 with the Indian group, of approximately 0.08 with the Bangladeshi group, and values of between 0.02 and 0.05 for all other groups. These results suggest that the effects of both occupational and environmental exposure are greater on COVID-19 infections for those living in neighbourhoods with a greater share of the Pakistani population.

Bringing this together, the Poisson model of infection rates (Eq. 4) is refitted to recalculate the local index of infection, this time conditional on additional co-variates for the log of the trimmed mean house price, mean-centred per town or city, residential density, the percentages of the neighbourhood populations employed as key workers, and the percentages of the adult population in various age groups from 18 to 80 and above. Most often it is average house price and residential density that are significant in their effect on the infection rate, at a 95 per cent confidence interval—house price in 79% of the weeks, density in 69%. Higher residential density is positively correlated with infections; increased house price negatively so.

**Fig. 5:**
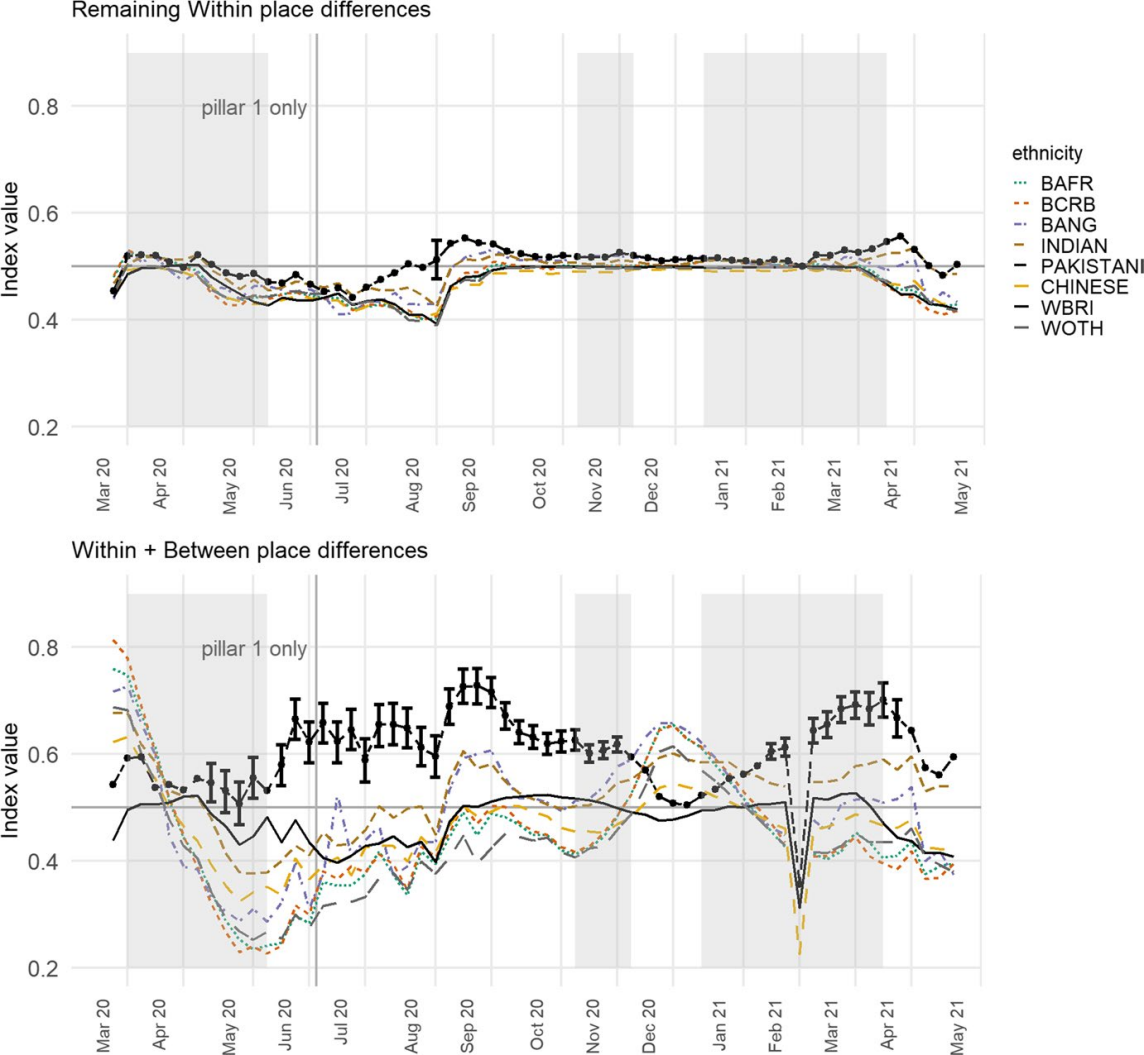
Showing the Index of Local Exposure values before and after the inclusion of the additional predictor variables

Of interest is what differences remain in the index of local exposure values, calculated from the remaining variations in the log infection rates for the towns and cities having included the additional co-variates. Whilst the Pakistani group still has the highest value for 40 of the 61 weeks (66%), with the Indian group highest for 11, the Bangladeshi group for 8, and the Black Caribbeans for 2, it is clear in Fig. 5 that the differences are not distinct. However, if we track back and return the differences between (not just within) towns and cities to the index, then the differences between the groups are evident again, with distinct differences between the Pakistani and groups on 38 of the 61 occasions (62%). Whilst the social, environmental and demographic variables are successful in explaining the differences within the towns and cities, geographical differences remain between them that disproportionately affect the neighbourhoods in which Pakistanis are living.

## Conclusion

This paper has developed an index of exposure using an underlying Bayesian Poisson model of the COVID-19 infection rates for neighbourhoods in English major towns and cities. The index measures how exposed various ethnic groups are to COVID-infected residential neighbourhoods and can be modified to allow for the broad-scale geography of the disease, focusing, instead, on the localised highs and lows of who is exposed. The results support the view that different ethnic groups have experienced different outcomes during the waves of the virus. Higher index values are true of Black groups in the initial weeks of the pandemic when the rates of infection are highest in London. They become more greatly characteristic of Pakistani and, to a lesser extent, Bangladeshi and Indian groups as the prevalence of the disease shifts to other towns and cities that include Oldham, Bradford, Blackburn, Manchester, Rochdale, Northampton and Leicester.

On February 18, 2021, the Department of Health and Social Care published interim findings from a study, showing that “large household size, living in a deprived neighbourhood, and areas with higher numbers of Asian ethnicity individuals were associated with increased prevalence [of COVID-19]” (Department of Health & Social Care, 2021). This accords with our findings, with the caveat that we observe the higher exposure values for the Pakistani group not solely associated with the least wealthy neighbourhoods but remain evident when the index is stratified into deciles based on property prices. This may be because the neighbourhoods, the Middle Level Super Output Areas, are internally heterogenous regarding their housing stock. However, we suspect a more structural explanation: ethnic inequalities in housing mean that members of the Pakistani group are living in higher residential density areas and, with the exception of the Bangladeshi group, are more likely to be living in overcrowded and/or intergenerational households.

Shankley and Finney (2020) observe that ethnic inequalities in housing “stem from the particular settlement experiences of postwar migrants to the UK in terms of the locations and housing areas afforded to them […] consolidated by dramatic changes to the UK’s housing landscape over recent decades [that have] exacerbated housing disadvantage for minorities” (p.149). They note the practices of discrimination and racism that existing in housing, the reduction in the social housing stock available and problems of affordability and financial risk. The overarching issue of excessive housing costs (rented or purchased), relative to income, adds to financial pooling, sharing and to overcrowding in many towns and cities.

Housing inequalities are not the only explanation for why particular ethnic groups live, disproportionately, in COVID-infected neighbourhoods. The *Doreen Lawrence Review* also identifies occupational exposure, which we find evidence for too. Both are linked to other sub-national, socio-economic inequalities including the imbalanced nature of regional economies and employment structures in the UK (ONS, 2020e). Writing in *The Guardian*, Dorling (2020) picks up these themes, arguing that the key to understanding the geography of COVID-19, “is the underlying social and economic geography of England. To understand the changing medical geography of this pandemic, you must first understand how the country lives and works.” In policy terms, what we have is not simply a pandemic, with biological origins but a syndmeic, with social origins too (Horton, 2020). It follows that long-term resilience is not just a matter of mass vaccination. It requires a shot in the arm to tackling socio-spatial inequalities and their causes, including the unaffordability of housing (Mean, 2018).

## Footnotes

1 A reason for the usually higher mortality amongst White groups is that the White British population is, on average, older.

2 Infection rates were also at their highest during the second wave, peaking, at a UK rate of 600 cases per 100,000 at the end of the first week of January.

3 Early releases of the data were subsequently revised by the Government, first to harmonise them and to remove duplicates, and, secondly, to assign people more accurately to their current residential address. This raised the numbers around Universities as students were located to their term-time address, which may differ from the one on their NHS record because the latter can be a parental address if they are registered to a GP ‘back home’.

4 It uses stats::loess() in R with a span parameter of 0.5.

5 The delivery point information is from Ordnance Survey Code-Point data.
